# Associations between birth kit use and maternal and neonatal health outcomes in rural Jigawa state, Nigeria: A secondary analysis of data from a cluster randomized controlled trial

**DOI:** 10.1371/journal.pone.0208885

**Published:** 2018-12-26

**Authors:** Jessica Leight, Vandana Sharma, Willa Brown, Laura Costica, Fatima Abdulaziz Sule, Martina Bjorkman Nyqvist

**Affiliations:** 1 Economics Department, American University, Washington, D.C., United States of America; 2 Harvard T.H. Chan School of Public Health, Cambridge, MA, United States of America; 3 Abdul Latif Jameel Poverty Action Lab, Massachusetts Institute of Technology, Cambridge, MA, United States of America; 4 Planned Parenthood Federation of Nigeria, Abuja, Nigeria; 5 Economics Department, American University, Washington D.C., United States of America; 6 Stockholm School of Economics, Stockholm, Sweden; London School of Hygiene and Tropical Medicine, UNITED KINGDOM

## Abstract

**Background:**

The burden of maternal and neonatal mortality remains persistently high in Nigeria. Sepsis contributes significantly to both maternal and newborn mortality, and safe delivery kits have long been promoted as a cost-effective intervention to ensure hygienic delivery practices and reduce sepsis. However, there is limited evidence on the effectiveness of home birth kit distribution by community health workers, and particularly the impact of this intervention on health outcomes. This paper reports a secondary analysis of data from a cluster randomized trial in rural northern Nigeria in which birth kits were distributed by community health workers to pregnant women in their homes, analyzing non-experimental variation in receipt and use of birth kits. More specifically, associations between pregnant women’s baseline characteristics and receipt and use of birth kits, and associations between birth kit use, care utilization and maternal and newborn outcomes were assessed.

**Methods and findings:**

Baseline, post-birth and endline data related to 3,317 births observed over a period of three years in 72 intervention communities in Jigawa state, Nigeria, were analyzed using hierarchical, logistic regression models. In total, 140 women received birth kits, and 72 women used the kits. There were no associations between baseline demographic characteristics, health history, and knowledge and attitudes and receipt of a kit, suggesting that community health workers did not systematically target the distribution of birth kits. However, women who used the kit reported reduced odds of past pregnancy complications (OR = 0.44, 95% CI: 0.19-1.00) as well as significantly higher odds of feeling generally healthy at baseline (OR = 2.00, 95% CI: 1.06-3.76), of exposure to radio media (OR = 1.97, 95% CI: 1.21-3.22), and of perceiving themselves as having a low-risk pregnancy (OR = 3.05, 95% CI:1.39-6.68). While there were no significant associations between birth kit use and facility based delivery, skilled birth attendance or post-natal care, women who used a kit exhibited significantly lower odds of completing four or more ANC visits (adjusted OR = 0.39, 95% CI: 0.18-0.85) and significantly higher odds of reporting prolonged labor (adjusted OR = 4.75, 95% CI: 1.36-16.59), and post-partum bleeding (adjusted OR = 3.25, 95% CI: 1.11-9.52).

**Conclusions:**

This evidence suggests that use of birth kits is low in a rural population characterized by minimal baseline utilization of maternal and neonatal health services, and the use of birth kits was not associated with reductions in maternal or neonatal morbidity. While further research is required to understand how the effectiveness of birth kits may be shaped by the mechanism through which women access and utilize the kits, our findings suggest that the provision of kits to women outside of the formal health system may be associated with increased risk of adverse outcomes.

## Introduction

Despite increasing attention and commitments by policymakers globally to the goal of reducing maternal and neonatal mortality, both have remained stubbornly high in sub-Saharan Africa. In Nigeria, the neonatal mortality rate (NMR; number of newborn deaths per 1,000 live births) was roughly stagnant between 1990 and 2013, and the burden of neonatal mortality remains higher in Nigeria vis-a-vis other sub-Saharan African countries [1]. Moreover, Nigeria is one of six countries that jointly constitute more than 50% of global maternal deaths, and reductions in the maternal mortality ratio in Nigeria (MMR; number of maternal deaths per 100,000 live births) have been slow and inconsistent.

In addition, both MMR and NMR show geographical variation, and the highest levels are observed in the northern part of the country. The MMR in Jigawa state, a rural state in northern Nigeria that is the focus of this analysis, is estimated to be around 1,012 per 100,000 live births [2], compared to 576 per 100,000 at the national level [3]. The NMR in the region of northwest Nigeria is 42 per 1,000 live births, compared to a nationwide average of 37 [1]. These high mortality rates partly reflect low rates of facility delivery and skilled birth attendance, estimated to be 6.7% and 7.6% respectively in Jigawa [3], as well as challenges in appropriate recognition of complications and care-seeking by pregnant women and their families [4].

Sepsis contributes significantly to both maternal and neonatal deaths. The World Health Organization (WHO) estimates that 10.7% of maternal deaths [5] and 30% of newborn deaths are due to infections contracted during birth [6], and emphasizes the importance of the “six cleans” in reducing infection risk to mothers and newborns, including clean hands, a clean perineum, a clean delivery surface, a clean cord and tying instruments, and a clean cloth for drying [7, 8]. Both governmental and non-governmental stakeholders have highlighted the potential of birth kits (also known as safe delivery kits or clean delivery kits) as a low-cost intervention to enhance hygienic practices before, during and after delivery. More specifically, the WHO argues that kit use improves standards of care in home deliveries [6], and in deliveries in facilities that lack the capacity to sterilize equipment [9]. While the composition of birth kits and the mechanism by which health workers and women access them vary in different contexts, broadly speaking the kits comprise a package of sterile materials including a plastic sheet, soap, sterile liquid, a razor blade, cotton wool, gloves, and string.

Despite these recommendations, there is limited evidence around the impact of clean delivery kits on health outcomes, including neonatal sepsis and neonatal mortality [10–12]. The most recent systematic review of safe birth kit research found that only 18 studies reported results of an intervention including birth kits; all of these were observational studies except for one randomized controlled trial, and almost all were unable to separately identify the effects of birth kits compared to other interventions [10].

Two recent studies presented non-experimental evidence that women who used birth kits distributed by a primary health center in Egypt were significantly less likely to experience puerperal sepsis, and their neonates were less likely to develop cord infection [13, 14]. A quasi-experimental evaluation of an intervention distributing safe birth kits via primary clinics in Tanzania also reported significant reductions in neonatal cord infection and maternal puerperal sepsis [15, 16]. Other recent work found that utilization of birth kits was correlated with reduced neonatal mortality at three sites in South Asia [17], but not with a reduction in the risk of maternal death [18]. In general, there is limited evidence around the associations between safe birth kits and maternal health service use. However, one RCT conducted in Zambia reported that distribution of “mama kits” containing only a cloth, diapers and blanket to women delivering in a facility (and only these women) increased the rate of facility delivery [19].

Conceptually, there are at least two important factors that may limit the effectiveness of birth kits. First, utilization rates of the kits themselves may be low outside of formal health care settings; the existing evidence almost exclusively focuses on interventions in which birth kits are distributed to health facilities or skilled birth attendants [10, 12], although an estimated 52 million women give birth at home without a skilled provider each year [20]. In what Hart deems the law of inverse care, women who are most vulnerable are generally characterized by the lowest rates of service utilization [21]. Second, women who have access to safe delivery kits may regard them as a substitute for other forms of formal health care, including antenatal (ANC), delivery and postnatal care, and risk adverse consequences of this reduced utilization. This may be of particular concern in populations characterized by low levels of human capital and access to health-related information. Although Hundley et al. highlight the possibility of a substitution effect in their review, to our knowledge there are no studies that examine this important question in a developing country setting [12].

The objective of this paper is to examine associations between baseline characteristics and birth kit use and receipt, as well as birth kit use and care utilization and maternal and neonatal outcomes, by conducting a secondary analysis of data from a cluster-randomized controlled trial (cRCT) implemented in Jigawa, Nigeria. In the main trial, birth kits were distributed by community health workers to pregnant women in their homes in one experimental arm. Given that the experimental design entailed the assignment of pregnant women to receive a birth kit independent of their interactions with the health system, their planned delivery location, or their desire to use the birth kit, we are able to assess whether women in a low-resource context with limited access to care use a birth kit, use it effectively, and demonstrate any improvement in health outcomes. Importantly, the paper fills a significant gap in the literature by analyzing the effects of birth kits when distributed by community health workers directly to pregnant women.

## Materials and methods

This study utilizes data from a cRCT of community-based interventions designed to reduce maternal and newborn mortality conducted in Jigawa state, Nigeria. Jigawa is located in northwestern Nigeria and is characterized by extremely poor baseline health outcomes, particularly for women and neonates. The region has also been exposed to ongoing violence linked to the Boko Haram conflict. The cRCT was implemented by the Abdul Latif Jameel Poverty Action Lab (J-PAL) in partnership with the Planned Parenthood Federation of Nigeria (PPFN) and was designed to evaluate three interventions: training local women as Community Resource Persons (CoRPs) to provide health education to pregnant women and their families; deploying the CoRPs in conjunction with the distribution of safe birth kits to pregnant women; and deploying the CoRPs in conjunction with community dramas to change social norms around maternal health. This paper does not report the primary experimental effects of the interventions; these effects are reported in a separate, forthcoming paper.

### Ethical approval

Ethical approvals for the cRCT analyzed here were provided by the Massachusetts Institute of Technology (MIT) and the Operational Research Committee (ORAC) of the Ministry of Health in Jigawa state, Nigeria. ORAC serves as the institutional review board for human subject research conducted within Jigawa state. The cRCT protocol is registered at ClinicalTrials.gov under the registration number NCT01487707. All data collection was conducted in Jigawa, a region with low female literacy.

All respondents provided written consent using paper consent forms, and the consent process was documented in the electronic data system by enumerators. In addition, consent was sought from the parents or guardians of any non-emancipated minors included in the sample. These consent processes were approved by the relevant Institutional Review Boards.

### Study population and interventions

Jigawa state had a population of 4.3 million during the 2006 census, and includes 27 local government areas (LGAs); 80% of the population lives in rural areas [22]. The state is characterized by generally low rates of maternal health services utilization and poor baseline health outcomes, including the third lowest rate of facility based delivery in the country (6.7% vs 35.5% nationally), and the fourth lowest percentage of fully vaccinated children 12-23 months of age (4% vs 25% nationally) [3]. In response to the observed low rates of facility delivery, the Nigerian government rolled out the Midwives Service Scheme (MSS) in 2009, recruiting midwives to be deployed to government primary health centers (PHCs) in rural communities to provide 24-hour maternity care [23].

In northern Nigeria, key stakeholders in the health sector hypothesized that the expansion of the MSS alone might not lead to enhancement of maternal and child health outcomes given the observed low rates of utilization of maternal health care ex ante. Accordingly, our partner organization, the Planned Parenthood Federation of Nigeria (PPFN), proposed to implement three community-based interventions involving health educators deemed community resource persons or CoRPs, designed to stimulate utilization of these newly available maternal health services and thus enhance maternal and neonatal health [24]. These interventions were evaluated using a cluster randomized controlled trial with four experimental arms.

In the first experimental arm, CoRPs services were rolled out to sampled villages; CoRPs are local women between 20 and 45 years of age recruited by PPFN in conjunction with local ward health committees. They received a one-week training and were mandated to provide information on antenatal care, nutrition in pregnancy, identification of danger signs, birth preparedness, labor and postnatal care, breastfeeding, immunization, birth registration, and family planning over a series of six home visits to pregnant women. PPFN described CoRPs as serving as “a bridge crucial to foster trust, confidence and acceptance between the midwives and their clients and to ensure effective communication between the two groups”. The CORPs also received a small stipend (2000 naira a month, or approximately $5).

In the second experimental arm, CoRPs were provided with birth kits to distribute to pregnant women in their third trimester, in conjunction with the provision of instructions around how to use the kit. The kits included a plastic sheet for the woman to lie on during delivery, surgical gloves for a birth attendant to utilize, a sterile razor and cord clamps to cut and tie the umbilical cord, methylated spirit to clean the umbilical stump, clean gauze, swabs and perineal pads to be used by the mother after birth, a gallipot, a mechanical suction tube to clear secretions from the baby’s airways, and a wrapper and diapers for the use of the mother and baby immediately after birth [25]. All materials were packaged in a single sterile unit.

In the third experimental arm, in addition to the CoRPs intervention, PPFN also conducted community drama activities in order to promote the importance of safe motherhood at the community level. The fourth experimental arm served as the control arm, in which PPFN did not provide any new services. Women in control communities continued to have access to standard health services provided at MSS clinics.

The RCT design entails randomization at the community level, given that all three interventions are subject to significant within-community spillovers. In total, 96 clusters in 24 local government areas (LGAs) were included. All clusters were located within 20 kilometers of a MSS PHC, and each cluster included 75 respondents enrolled at baseline. In addition, neonatal deaths were tracked for the full cluster population (approximately 3,000 individuals per cluster).

Baseline power calculations estimated the cRCT had 90% power to detect a 24% decrease in neonatal mortality and a 23% decrease in maternal morbidity using a one-tailed test (*α* = .1, *K* = .2); this is assuming a baseline neonatal mortality rate of 47 per 1,000 and a baseline maternal morbidity rate of 35%, and attrition of less than 5% [26]. Attrition was assumed to be low given that data would be collected about pregnancies at two points in time: shortly after the birth, and again at endline. Given limited ex ante evidence on intracluster correlation for the outcome variables of interest in the Nigerian context, the power calculations drew on published estimates of *k* for perinatal indicators in other developing countries as well as methodological guidance for sample size calculation in cluster randomized trials [27, 28].

### Data collection and management

The main trial included three phases of data collection: baseline, continuous data collection, and endline. Data were collected by a team of trained female Hausa-speaking enumerators, recruited from Jigawa state; at baseline, the team also included some individuals recruited from neighboring states. Enumerators received a minimum of two weeks training focusing on survey administration, ethics, and techniques of anthropometric measurement. Enumerators were supervised by a team of four on-the-field supervisors who were responsible for overseeing data quality by directly observing enumerators and conducting backcheck surveys. Data were collected on smart phones using SurveyCTO, and surveys were administered in Hausa.

The baseline survey (N = 7,069) was conducted between December 2011 and May 2012 using a 15% random sample of all enumerated households including a woman of reproductive age (between 15 and 49); if more than one woman of reproductive age was present in a sampled household, one woman was randomly selected utilizing an on-the-field randomization protocol. The baseline survey included information about household composition and socioeconomic characteristics, birth history, utilization of health services for pregnancies within the preceding 24 months, contraceptive utilization, the respondent’s health and the health of any infant children, and health knowledge and attitudes. In addition, anthropometric measurements were collected for the respondent’s children under the age of two (i.e., children born during the intervention period).

Following the baseline, continuous monitoring of pregnancies via a RapidSMS surveillance system was initiated between November 2013 and November 2015. Female community members were recruited and trained to monitor pregnancies and vital events among baseline respondents, and were assigned to send a simple SMS to the survey team when a birth was observed in baseline households, and to report deaths of women and infants in any household. The SMS messages were then redirected to an enumerator who had the responsibility of identifying the household and administering questionnaires within 3 days and at 28 days after birth. For deaths of women of reproductive age, verbal autopsies were conducted to determine the cause of death.

The three-day survey included questions about utilization of antenatal care, the mother’s health during pregnancy, the delivery itself, and the mother’s and infant’s health since birth; the infant’s weight and length were also measured. The 28-day survey included questions about attitudes toward utilization of maternal health care, maternal and neonatal morbidity in the first month, and infant health practices; again, infant weight, length and mid-upper arm circumference (MUAC) were measured. In addition, an audit of all baseline households was conducted at the halfway point of continuous data collection in which all baseline households were revisited. The enumerators were mandated to pose a brief series of questions about births in the household, and to collect additional information from any births that had previously been missed. An endline survey with the baseline sample was conducted between February 2016 and July 2016.

Attrition in the endline was 9.8%. However, some women who were not surveyed at endline due to migration or divorce had been represented in earlier surveys (post-birth surveys or the audit). Accordingly, the rate of attrition from any follow-up data collection was only 7.8% of the original sample.

### Statistical methods

This paper reports summary statistics on receipt and use of birth kits in the target population, and uses hierarchical logistic regression to analyze associations between baseline characteristics of respondents and receipt and use of birth kits, as well as associations between use of birth kits and outcomes during pregnancy and delivery. The analysis focuses on the sample “as treated” and is complementary to an intention-to-treat analysis analyzing experimental evidence around the primary effects of the intervention, reported in a separate, forthcoming paper. More specifically, an “as treated” analysis is utilized here given that an intention-to-treat analysis cannot provide evidence about the correlations between birth kit use and health outcomes.

Receipt and use of birth kits varied at the individual level even within clusters assigned to receive birth kits. Accordingly, individual-level variation rather than cluster-level variation was employed, using data from women who reported a birth during the evaluation period in any of the three intervention arms; this was a sample of 3,317 women. For the analysis of birth kit use, the sample was restricted to women who reported receipt of a birth kit from a CoRP.

Given that there is presumably correlation in delivery outcomes within clusters, the analysis accounted for this intra-cluster correlation by estimating logistic regressions including clustered standard errors. Data were analyzed using Stata 14.2 (Stata Corp, College Station, TX), and the Stata command “logit” was employed, using the “cl” option. More specifically, logit models were estimated iteratively employing maximum likelihood estimation, and sandwich standard errors were employed to conduct inference given an arbitrary structure of within-cluster correlation of errors [29].

Binary variables capturing birth kit receipt and use were constructed as follows: “birth kit receipt” was defined as equal to one if the respondent reported receipt of a birth kit from a PPFN CoRP. “Birth kit use” was defined as equal to one if the respondent reported using the birth kit during her delivery. The baseline characteristics assessed included demographic characteristics, health history, utilization of health services, health knowledge, knowledge of pregnancy-related complications, perceptions of risk in pregnancy and childbirth, and household dynamics, as described in more detail below.

#### Baseline characteristics

Demographic characteristics included variables capturing age, ethnicity, household structure, educational background, birth parity, and a wealth index. Birth parity was defined as the number of births (including stillbirths) reported by the respondent; it was converted to a categorical variable for parity zero, parity one, and parity 2+. The household wealth index was computed by principal component analysis from four binary variables capturing whether the household reports a solid roof, a solid floor, access to a latrine and a house constructed from solid materials.

Variables capturing baseline health history and health care utilization included the respondent’s history of miscarriage, stillbirths, infant deaths, and pregnancy complications, and antenatal care utilization, skilled attendance at birth, and utilization of postnatal care as reported for her last birth. Additional variables captured baseline health knowledge, including radio exposure, coded as equal to one for women who report listening to the radio at least once a week, and awareness of tuberculosis, HIV/AIDS, and the importance of exclusive breastfeeding. Variables capturing baseline knowledge of pregnancy-specific complications included the number of labor and delivery complications, danger signs, and postpartum complications identified by the respondent.

The respondent’s perceptions of risk in pregnancy and delivery were measured using the following variables: whether she believes maternal death to be preventable, whether she is confident she will not encounter challenges in delivery, and three binary variables representing whether the respondent correctly identified the riskier scenario in a series of questions posed about pregnancy, delivery, and the post-partum period. Based on the number of correct responses, women were assigned to one of three categories of risk knowledge (low, medium and high knowledge, corresponding to one, two, and three correct responses, respectively). Finally, baseline household dynamics were captured by the husband’s educational and occupational characteristics and household decision-making patterns. It is important to note that the sample for some regressions is more limited if certain variables are not reported at baseline; in particular, respondents who had not reported a birth in the 24 months prior to baseline do not report baseline care utilization.

#### Pregnancy outcomes

In the final part of the analysis, associations between birth kit use and pregnancy outcomes (health care utilization, health practices, neonatal and maternal health outcomes, and under-two anthropometric measurements) were analyzed. The variables of interest are defined in Table 1. Pregnancy outcomes were selected based on a review of the relevant literature on the impact of birth kits and characteristics predictive of maternal and neonatal care utilization.

**Table 1:** Pregnancy outcomes of interest.

Variable	Description
**Care utilization**	
Utilized antenatal care	Respondent attended at least 1 antenatal visit from a skilled provider
Attended ≥ 4 ANC visits	Respondent attended 4 or more antenatal visits from a skilled provider
Utilized ANC in first trimester	Respondent attended ANC in first trimester
Received ≥ 50%ANC services	Respondent received more than 50% of available antenatal services; services included body weighing, height measurement, blood pressure check, blood test, urine test, stomach height measurement, listening to fetus heart, internal check, HIV test, advice about proper nutrition, information on indications of complication, advice on what to do in case of complication
Received ANC at PHC or hospital	Respondent attended ANC at a higher-level facility (PHC, hospital)
Received tetanus vaccine	Respondent received tetanus vaccine during ANC
Received iron folic pills	Respondent received iron folic pills during ANC
Utilized care given complications	Respondent used care from a skilled provider if she experienced a pregnancy complication (Missing if no complication reported)
Facility birth	Respondent delivered in a health facility
Delivered at home alone	Respondent delivered at home, with no other individual present
Delivered at home accompanied	Respondent delivered at home, accompanied by another individual
Skilled attendance at birth	Delivery was attended by a skilled provider
Utilized postnatal care	Respondent received postnatal care within two months of delivery
**Health practices**	
Developed birth plan	Respondent developed a birth plan prior to delivery
Husband present at ANC	Respondent reported husband attended ≥1 ANC visit
Husband present at delivery	Respondent reported husband present at delivery
Complementary feeding in first 3 days	Respondent provided liquid or food to infant in first 3 days of life
**Maternal and neonatal morbidity**	
Pregnancy—swelling; fatigue; high BP; other	Respondent reported complication during most recent pregnancy
Delivery—bleeding; prolonged labor; headache / blurred vision / high BP	Respondent reported complication during most recent delivery
Post-partum—bleeding; swelling; fever; abdominal pain	Respondent reported complication during most recent post-partum period (60 days post-birth)
Neonatal—rash, fever	Respondent reported infant experienced specified complication within first 60 days of life
**Under-two anthropometrics**	
Underweight	Weight-for-age is < 2 SD below mean of WHO reference population
Stunted	Height-for-age is < 2 SD below mean of WHO reference population
Low MUAC-for-age	MUAC-for-age is < 2 SD below mean of WHO reference population

Care utilization variables included whether the respondent utilized antenatal care and additional questions describing her care utilization pattern (if more than four antenatal visits were conducted, if ANC was initiated in the first trimester, if the respondent received certain ANC services including a tetanus vaccine and iron folic pills, and if she received ANC at a PHC or hospital), whether the respondent delivered in a facility, whether she delivered at home (alone or accompanied), whether a skilled attendant was present at the birth, and whether she utilized postnatal care. Variables capturing health practices included whether the respondent developed a birth plan, whether the husband was present at ANC and/or delivery, and whether complementary feeding was initiated in the first three days. Variables capturing maternal and neonatal morbidity included a series of questions around whether the respondent experienced enumerated symptoms during pregnancy, delivery or post-partum, and whether the respondent reported symptoms of illness for the infant in the first 60 days of life. In addition, three binary variables were constructed capturing whether the respondent’s children under two at endline are underweight, stunted or characterized by low MUAC-for-age, defined as weight-for-age, height-for-age, or MUAC-for-age more than two standard deviations below the mean of the WHO reference population.

For these variables, we preferentially utilized data reported in the surveys conducted three and 28 days after birth; if these surveys were missing, we drew on parallel data reported in the audit survey or the endline. Given the long follow-up period and the challenges posed by data collection in a remote and conflict-affected region, we benefited from utilizing these various complementary sources of data to obtain maximum information about our sample of interest. Again, this analysis was restricted to women who reported they received a birth kit.

In these specifications, we also considered variables that may confound or modify these associations. The confounding variables included were dummy variables for age categories, Hausa ethnicity, marital status, polygamous status, ever attended school, literacy, birth parity status (parity zero, parity one, and parity two or higher), and assignment to the birth kits arm. The specifications examining maternal and neonatal morbidity and under-two anthropometrics also adjusted for variables capturing utilization of maternal health care: whether the respondent utilized antenatal care, whether she delivered in a facility, and whether the delivery was attended by a skilled provider. The confounding variables were chosen based on a review of the literature around demographic variables predictive of birth kit utilization and maternal health outcomes.

## Results

### Summary statistics around use of birth kits

**Fig 1:**
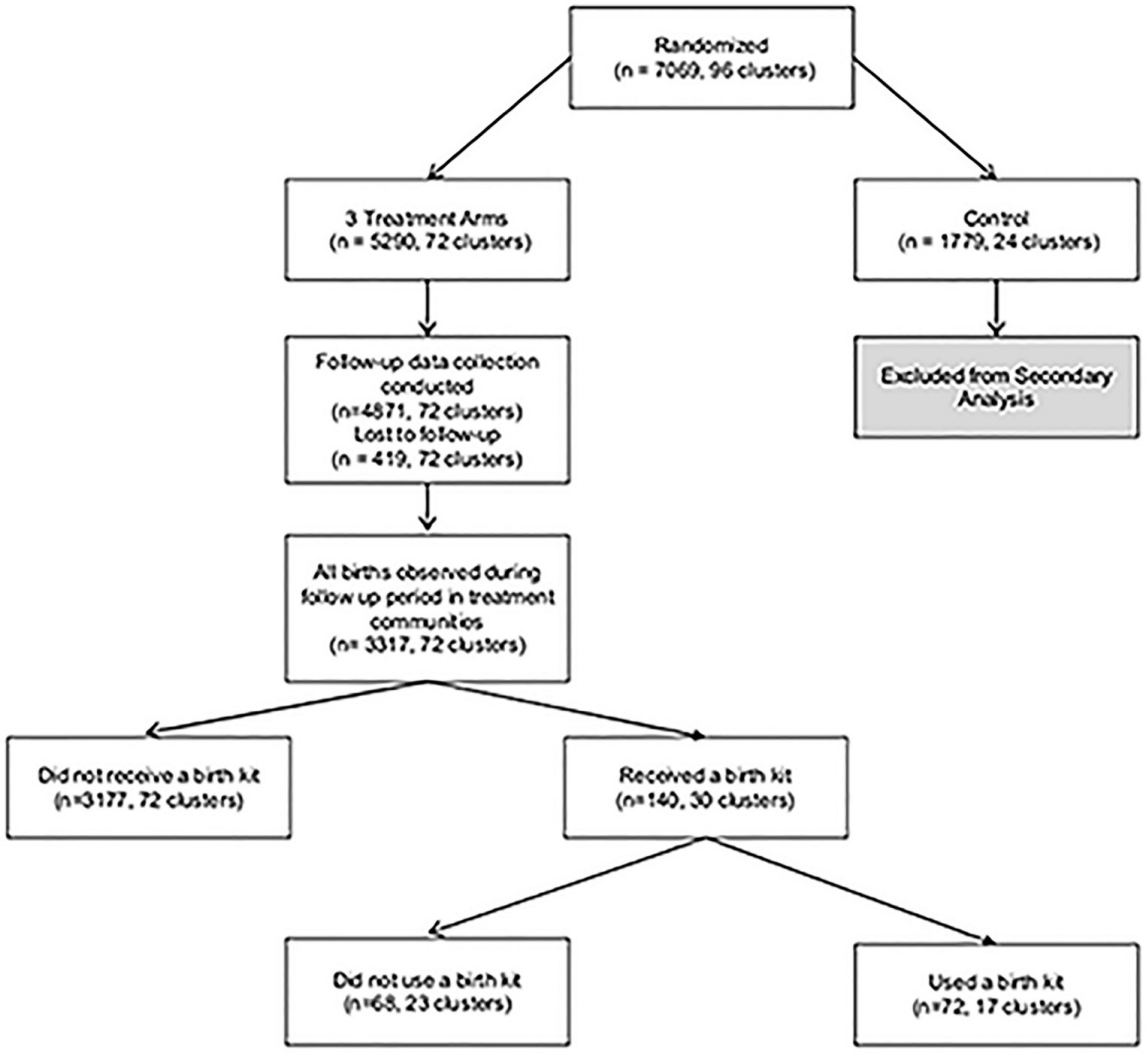
Flowchart depicting respondents included in analysis.

Fig 1 depicts the flow of participants sampled for this secondary analysis. In total, 7,069 women in 96 clusters were initially sampled for inclusion in the cRCT; 72 clusters comprising 5,290 women were randomly assigned to one of the three intervention arms, while 24 clusters comprising 1,779 women were assigned to the control arm. The latter are excluded from this analysis. In the three intervention arms, follow-up data was collected from 4,871 women, while 419 were lost to follow up. Among those respondents who were observed in follow-up data, 3,317 births during the follow up period were reported. (The sample of reported births includes reported stillbirths, but data on miscarriages was not collected.) Within the subsample of women reporting births, 140 women in 30 clusters reported receipt of a birth kit from PPFN CoRPs, and 72 of these women in 17 clusters reported using the birth kit.

Table 2 provides further detailed summary statistics around use of birth kits. Only 9.9% of pregnant women in the birth kits arm reported receiving a kit; thus intervention penetration, defined as the percentage of eligible respondents assigned to receive birth kits who in fact received kits, was 9.9%. In addition, 27 women in treatment communities assigned to receive other interventions reported receiving a kit.

**Table 2:** Summary statistics on birth kit use at the individual level.

Exposure to birth kits	N	%
Received a kit: Birth kits arm		
Yes	113	9.9%
No	1027	90.1%
Received a kit: Other intervention arms		
Yes	27	1.2%
No	2150	98.8%
Recipient knows how to use kit		
Yes	102	74.3%
No	38	25.7%
Recipient names at least one object in the kit		
Yes	93	66.4%
No	47	33.6%
Recipient use of kit		
Used	72	51.4%
Retains in possession	61	43.6%
Discarded	1	0.01%
Gave it to friends	3	2.1%
Unknown	3	2.1%

Conditional on receiving a birth kit, knowledge was relatively high. 74.3% (N = 102) of women who received a kit reported knowing how to use it, and 66.4% (N = 93) could correctly name at least one object included in the kit. Use rates were lower; only 51.4% (N = 72) report they used the kit in their most recent delivery. The majority of respondents who did not use the kit reported they were still in possession of it. Only one woman reported she discarded the kit, while three respondents reported passing the kit to a friend.

### Baseline characteristics and kit receipt

Tables 3 and 4 present associations between birth kit receipt and baseline characteristics. Broadly, the sample is characterized by low levels of human capital and low rates of utilization of formal health care. The average respondent was approximately age 26 at baseline, married at age 15, and had three living children. Nearly 30% of respondents identified their households as polygamous. Only about 20% of respondents ever attended school, and 10% reported that they are literate in Hausa; slightly over 40% reported living in a home with a solid roof, but less than 10% reported living in a home with a solid floor. While rates of antenatal care usage reported in the most recent pregnancy were relatively high (the average number of antenatal visits is four), less than 10% of women stated that their last delivery was in a health facility. At baseline, 47% of women reporting a previous birth had experienced a child death, and 10% had experienced a stillbirth.

**Table 3:** Associations between baseline characteristics and birth kit receipt at the individual level.

	N (%)	N (%)	OR	95% CI
	Did not receive (n = 3177)	Received a kit (n = 140)		
	**Panel A: Baseline socioeconomic characteristics**			
Age 16–19	737 (23.2)	31 (22.1)	0.96	[0.68–1.37]
Age 29–29	1458 (45.9)	66 (47.1)	1.09	[0.75–1.57]
Age 30 +	982 (30.9)	43 (30.7)	0.94	[0.64–1.37]
Hausa	2645 (83.3)	120(85.7)	1.17	[0.61–2.24]
Marital status (first marriage)	2553 (80.4)	118 (84.3)	1.20	[0.68–2.12]
Household polygamous	923 (29.1)	47 (33.6)	1.19	[0.83–1.70]
Attended any school	615 (19.4)	28 (20.0)	1.00	[0.56–1.80]
Literate in Hausa	346 (10.9)	20 (14.3)	1.31	[0.69–2.48]
Parity zero	374 (11.8)	12 (8.6)	0.75	[0.42–1.36]
Parity one	477 (15.0)	24 (17.1)	1.23	[0.78–1.92]
Parity 2+	2326 (73.2)	10 4(74.3)	1.02	[0.69–1.50]
Age at marriage^†^	15.3 (1.9)	15.1 (1.5)	0.93	[0.82–1.06]
Number of children^†^	2.8 (2.2)	3 (2.3)	1.04	[0.97–1.11]
Wealth index^†^	0 (1.3)	0 (1.3)	1.05	[0.90–1.23]
	**Panel B: Baseline health history**			
Feels generally well	2055 (64.7)	85 (60.7)	0.86	[0.56–1.32]
Any miscarriage	344 (10.8)	12 (8.6)	0.76	[0.45–1.26]
Any stillbirth	434 (13.7)	19 (13.6)	0.97	[0.59–1.60]
Any death of infant (under 1)	1288 (46.4)	68 (54.0)	1.23	[0.87–1.73]
Any complication in last pregnancy	1468 (56.3)	69 (56.6)	1.04	[0.72–1.51]
	**Panel C: Baseline health utilization**			
Utilized antenatal care	1356 (52.0)	72 (59.0)	1.33	[0.91–1.93]
Attended ≥ 4 ANC visits	831 (31.9)	48 (39.3)	1.35	[0.85–2.13]
Utilized ANC in first trimester	278 (10.7)	16 (13.1)	1.25	[0.72–2.16]
Received 50%+ ANC services	601 (23.0)	37 (30.3)	1.39*	[1.01–1.91]
Received ANC at PHC or hospital	1273 (93.9)	66 (91.7)	0.92	[0.31–2.80]
Received tetanus vaccine	1174 (45.0)	58 (47.5)	1.08	[0.66–1.75]
Received iron folic pills	1392 (53.4)	74 (60.7)	1.34	[0.89–2.00]
Care utilization given complications	657 (50.2)	32 (49.2)	0.90	[0.56–1.43]
Facility delivery	179 (9.6)	10 (12.0)	1.44	[0.72–2.89]
Skilled attendance at birth	204 (11.0)	10 (12.0)	1.32	[0.65–2.65]
Utilization of postnatal care	510 (27.2)	21 (25.3)	0.87	[0.57–1.33]

The dagger symbol indicates continuous variables for which the mean is reported, rather than count data; the standard deviation is reported in parentheses. Asterisks indicate significance at the 5 (*), 1 (**), and .1 (***) percent level.

**Table 4:** Associations between baseline characteristics and birth kit receipt at the individual level.

	N (%)	N (%)	OR	95% CI
	Did not receive (n = 3177)	Received a kit (n = 140)		
	**Panel A: Baseline health knowledge**			
Listens to radio regularly	1302 (41.0)	67 (47.9)	1.33	[0.92–1.91]
Aware of TB	2126 (66.9)	99 (70.7)	1.07	[0.76–1.50]
Aware of HIV/AIDS	2632 (82.8)	124 (88.6)	1.37	[0.89–2.12]
Aware of MTCT	1233 (46.8)	67 (54.0)	1.29	[0.91–1.83]
Aware of HIV transmission via breastfeeding	1472 (55.9)	83 (66.9)	1.55*	[1.02–2.34]
Aware of birth control pill	1054 (33.2)	51 (36.4)	1.20	[0.82–1.78]
Aware infant should be immediately breastfed	1397 (44.0)	72 (51.4)	1.33	[0.89–1.97]
Aware of exclusive breastfeeding for one month	238 (7.5)	12 (8.6)	1.11	[0.65–1.89]
	**Panel B: Baseline knowledge of pregnancy–specific complications**			
Number of labor and delivery complications known ^†^	3.1 (3.1)	3.2 (3.3)	1.01	[0.94–1.09]
Number of danger signs known ^†^	3.0 (3.2)	3.1 (3.3)	1.01	[0.94–1.09]
Number of postpartum complications known^†^	2.6 (2.5)	2.7 (2.5)	1.01	[0.92–1.12]
	**Panel C: Baseline perceptions of risk in pregnancy and delivery**			
Believes maternal death to be preventable	357 (11.2)	19 (13.6)	1.14	[0.62–2.10]
Confident that she won’t have problems in delivery	1303 (41.0)	62 (44.3)	1.10	[0.69–1.74]
Relative risk: low knowledge	765 (24.1)	24 (17.1)	0.71	[0.39–1.29]
Relative risk: medium knowledge	238 (7.5)	8 (5.7)	0.71	[0.33–1.53]
Relative risk: high knowledge	2174 (68.4)	108 (77.1)	1.48	[0.82–2.67]
	**Panel D: Baseline household dynamics**			
Husband received education (secondary or higher)	504 (22.3)	35 (34.7)	1.98**	[1.20–3.28]
Husband occupation: agriculture	1550 (48.8)	76 (54.3)	1.22	[0.79–1.90]
Husband decides alone: finances	2700 (85.0)	112 (80.0)	0.76	[0.47–1.22]
Husband decides alone: children’s health	2136 (82.8)	101 (82.1)	0.97	[0.56–1.66]
Husband decides alone: antenatal care	2965 (93.3)	130 (92.9)	0.88	[0.44–1.78]
Husband assisted in most recent delivery	1025 (54.8)	46 (55.4)	0.98	[0.59–1.62]

The dagger symbol indicates continuous variables for which the mean is reported, rather than count data; the standard deviation is reported in parentheses. Asterisks indicate significance at the 5 (*), 1 (**), and .1 (***) percent level.

When comparing women who do and do not report receipt of a birth kit, women who received a kit exhibited significantly increased odds of receiving more than 50% of available ANC services in their most recent pregnancy (OR 1.39, 95% CI: 1.01–1.91, *p* < 0.05), of reporting awareness of HIV transmission via breastfeeding (OR 1.55, 95% CI: 1.02–2.34, *p* < 0.01), and of having a spouse who completed secondary school (OR 1.98, 95% CI: 1.20–3.28, *p* < 0.01), relative to women who did not receive a birth kit. Otherwise, women who received a birth kit were observed to have similar observable characteristics to women who did not receive a kit.

### Baseline characteristics and kit use

Tables 5 and 6 present associations between birth kit use and baseline characteristics, restricting the sample to the 140 respondents who reported receipt of a birth kit from PPFN. Women who used the kit were more likely to report that their current marriage was their first (OR 2.82, 95% CI: 1.19–6.66, *p* < 0.05) and that they felt generally well (OR 2.00, 95% CI: 1.06–3.76, *p* < 0.05), and were less likely to report a complication in a previous pregnancy (OR 0.44, 95% CI: 0.19–1.00, *p* < 0.05). Kit users were also significantly more likely to report utilization of antenatal care (OR 2.53, 95% CI: 1.27–5.04, *p* <.01) and receipt of iron folic pills (OR 2.58, 95% CI: 1.22–5.47, *p* < 0.05) in their most recent pregnancy.

**Table 5:** Associations between baseline characteristics and birth kit use at the individual level.

	N (%)	N (%)	OR	95% CI
	Did not use (n = 68)	Used a kit (n = 72)		
	**Panel A: Baseline socioeconomic characteristics**			
Age 16–19	16 (23.5)	15 (20.8)	0.80	[0.37–1.73]
Age 29–29	28 (41.2)	38 (52.8)	1.66	[0.86–3.19]
Age 30 +	24 (35.3)	19 (26.4)	0.66	[0.43–1.03]
Hausa	57 (83.8)	63 (87.5)	1.20	[0.37–3.89]
Marital status (first marriage)	53 (77.9)	65 (90.3)	2.82*	[1.19–6.66]
Household polygamous	22 (32.4)	25 (34.7)	1.06	[0.50–2.25]
Attended any school	11 (16.2)	17 (23.6)	1.60	[0.64–3.96]
Literate in Hausa	7 (10.3)	13 (18.1)	2.06	[0.56–7.52]
Parity zero	4 (5.9)	8 (11.1)	1.75	[0.41–7.42]
Parity one	11 (16.2)	13 (18.1)	1.07	[0.34–3.34]
Parity 2+	53 (77.9)	51 (70.8)	0.74	[0.32–1.69]
Age at marriage^†^	14.9 (1.6)	15.2 (1.5)	1.13	[0.88–1.45]
Number of children^†^	3.1 (2.2)	2.9 (2.3)	0.97	[0.85–1.11]
Wealth index^†^	0.0 (1.2)	0.1 (1.4)	1.21	[0.90–1.61]
	**Panel B: Baseline health history**			
Feels generally well	36 (52.9)	49 (68.1)	2.00*	[1.06–3.76]
Any miscarriage	7 (10.3)	5 (6.9)	0.76	[0.25–2.29]
Any stillbirth	9 (13.2)	10 (13.9)	0.98	[0.36–2.68]
Any death of infant (under 1)	38 (60.3)	30 (47.6)	0.57	[0.32–1.03]
Any complication in last pregnancy	40 (65.6)	29 (47.5)	0.44*	[0.19–1.00]
	**Panel C: Baseline health utilization**			
Utilized antenatal care	29 (47.5)	43 (70.5)	2.53**	[1.27–5.04]
Attended ≥ 4 ANC visits	20 (32.8)	28 (45.9)	1.66	[0.86–3.22]
Utilized ANC in first trimester	6 (9.8)	10 (16.4)	1.68	[0.49–5.71]
Received 50%+ available services	16 (26.2)	21 (34.4)	1.43	[0.78–2.60]
Received ANC at PHC or hospital	29 (100.0)	37 (86.0)	Missing—see table note	
Received tetanus vaccine	25 (41.0)	33 (54.1)	1.59	[0.78–3.26]
Received iron folic pills	30 (49.2)	44 (72.1)	2.58*	[1.22–5.47]
Care utilization given complications	19 (51.4)	13 (46.4)	0.77	[0.38–1.54]
Facility delivery	4 (9.1)	6 (15.4)	1.84	[0.46–7.30]
Skilled attendance at birth	4 (9.1)	6 (15.4)	1.84	[0.46–7.30]
Utilization of postnatal care	11 (25.0)	10 (25.6)	1.03	[0.33–3.23]

The dagger symbol indicates continuous variables for which the mean is reported, rather than count data; the standard deviation is reported in parentheses. The odds ratio and confidence interval cannot be estimated for this variable given that birth kit non-utilization perfectly predicts utilizing ANC at a PHC or hospital. Asterisks indicate significance at the 5 (*), 1 (**), and .1 (***) percent level.

**Table 6:** Associations between baseline characteristics and birth kit use at the individual level.

	N (%)	N (%)	OR	95% CI
	Did not use (n = 68)	Used a kit (n = 72)		
	**Panel A: Baseline health knowledge**			
Listens to radio regularly	27 (39.7)	40 (55.6)	1.97**	[1.21–3.22]
Aware of TB	45 (66.2)	54 (75.0)	1.47	[0.78–2.78]
Aware of HIV/AIDS	59 (86.8)	65 (90.3)	1.43	[0.46–4.48]
Aware of MTCT	31 (52.5)	36 (55.4)	1.21	[0.57–2.55]
Aware of HIV transmission via breastfeeding	35 (59.3)	48 (73.8)	2.16*	[1.12–4.19]
Aware of birth control pill	19 (27.9)	32 (44.4)	2.39*	[1.18–4.85]
Aware infant should be immediately breastfed	33 (48.5)	39 (54.2)	1.24	[0.54–2.82]
Aware of exclusive breastfeeding for one month	6 (8.8)	6 (8.3)	1.13	[0.35–3.62]
	**Panel B: Baseline knowledge of pregnancy–specific complications**			
Number of labor and delivery complications known ^†^	3.0 (3.2)	3.4 (3.4)	1.03	[0.94–1.11]
Number of danger signs known ^†^	2.9 (3.2)	3.2 (3.5)	1.02	[0.94–1.11]
Number of postpartum complications known^†^	2.7 (2.6)	2.6 (2.5)	0.99	[0.89–1.10]
	**Panel C: Baseline perceptions of risk**			
Believes maternal death to be preventable	9 (13.2)	10 (13.9)	1.24	[0.55–2.80]
Confident that she won’t have problems in delivery	22 (32.4)	40 (55.6)	3.05**	[1.39–6.68]
Relative risk: low knowledge	15 (22.1)	9 (12.5)	0.49	[0.21–1.13]
Relative risk: medium knowledge	1 (1.5)	7 (9.7)	7.03*	[1.09–45.29]
Relative risk: high knowledge	52 (76.5)	56 (77.8)	1.12	[0.59–2.11]
	**Panel D: Baseline household dynamics**			
Husband received education (secondary or higher)	11 (24.4)	24 (42.9)	2.76	[0.81–9.38]
Husband occupation: agriculture	38 (55.9)	38 (52.8)	0.91	[0.38–2.14]
Husband decides alone: finances	55 (80.9)	57 (79.2)	0.82	[0.31–2.15]
Husband decides alone: children’s health	50 (79.4)	51 (85.0)	1.41	[0.66–3.01]
Husband decides alone: antenatal care	65 (95.6)	65 (90.3)	0.42	[0.09–1.90]
Husband assisted inmost recent delivery	21 (47.7)	25 (64.1)	2.03	[0.97–4.26]

The dagger symbol indicates continuous variables for which the mean is reported, rather than count data; the standard deviation is reported in parentheses. Asterisks indicate significance at the 5 (*), 1 (**), and .1 (***) percent level.

In addition, there was a positive association between kit use and regular radio exposure (OR 1.97, 95% CI: 1.21–3.22, *p* < 0.01), and in general kit users demonstrated a higher level of general health knowledge; they were more likely to be aware of HIV transmission via breastfeeding (OR 2.16, CI: 1.12–4.19, *p* < 0.05), and more likely to be aware of the birth control pill (OR 2.39, 95% CI: 1.18–4.85, *p* < 0.05). Finally, in terms of attitudinal characteristics, we observe a positive association between kit use and a respondent’s confidence that she will not encounter challenges in a future birth (OR 3.05, CI: 1.39–6.68, *p* < 0.01) and a medium level of pregnancy risk knowledge (OR 7.03, 95% CI: 1.09–45.29, *p* < 0.05).

### Birth kit use and pregnancy outcomes

Panel A of Table 7 reports crude and adjusted odds ratios of the associations between use of a birth kit and patterns of health care utilization and health practices for the pregnancy during which the respondent received the kit. Birth kit use was significantly associated with decreased odds of achieving four or more antenatal care visits (aOR 0.39, 95% CI: 0.18–0.85, *p* < 0.05), as well as decreased odds of utilizing antenatal care at a primary health center or hospital (aOR 0.15, 95% CI: 0.06–0.42, *p* < 0.001). There was no significant association between birth kit use and facility based delivery, skilled birth attendance or postnatal care. Use of a birth kit was associated with increased odds that the respondent’s husband was present at the delivery (aOR 3.15, 95% CI: 1.07–9.25, *p* < 0.05), but also significantly increased odds of neonatal complementary feeding within the first three days of life (aOR 21.48, 95% CI: 5.08–90.83, *p* < 0.001).

**Table 7:** Associations between birth kit use and pregnancy outcomes at the individual level.

	N (%)	N (%)	OR	95% CI	OR	95% CI
	Did not use (n = 68)	Used a kit (n = 72)	Crude	Crude	Adjusted	Adjusted
	**Panel A: Care utilization and health practices**					
Utilized antenatal care	61 (89.7)	68 (94.4)	1.95	[0.50–7.63]	1.75	[0.50–6.08]
Attended ≥ 4 ANC visits	45 (78.9)	42 (60.9)	0.41*	[0.19–0.90]	0.39*	[0.18–0.85]
Utilized ANC in first trimester	10 (20.8)	11 (17.5)	0.80	[0.35–1.84]	0.70	[0.31–1.58]
Received 50%+ ANC services	36 (73.5)	40 (60.6)	0.56	[0.14–2.22]	0.59	[0.14–2.45]
Received ANC at PHC or hospital	40 (81.6)	27 (40.9)	0.16***	[0.06–0.39]	0.15***	[0.06–0.42]
Received tetanus vaccine	48 (96.0)	58 (84.1)	0.22	[0.04–1.18]	0.18	[0.03–1.11]
Received iron folic pills	48 (96.0)	61 (88.4)	0.32	[0.07–1.40]	0.22	[0.04–1.18]
Utilized care given complications	23 (76.7)	29 (80.6)	1.26	[0.36–4.40]	2.03	[0.78–6.81]
Facility birth	17 (25.4)	25 (34.7)	1.56	[0.67–3.67]	1.55	[0.64–3.74]
Delivered at home alone	17 (28.8)	21 (29.6)	1.04	[.41–2.61]	1.07	[.43–2.69]
Delivered at home accompanied	25 (42.4)	25 (35.2)	0.74	[0.31–1.77]	0.76	[0.32–1.84]
Skilled attendance at birth	11 (20.8)	21 (30.9)	1.71	[.67–4.37]	1.61	[.66–3.95]
Utilized postnatal care	6 (11.3)	11 (16.2)	1.51	[0.46–4.96]	1.32	[0.47–3.71]
Developed birth plan	7 (13.0)	6 (10.9)	0.82	[0.25–2.70]	0.93	[0.31–2.75]
Husband present at ANC	17 (31.5)	18 (32.7)	1.06	[.47–2.41]	1.15	[.4–3.27]
Husband present at delivery	7 (14.3)	15 (30.6)	2.65	[0.91–7.70]	3.15*	[1.07–9.25]
Complementary feeding in first 3 days	20 (39.2)	59 (89.4)	13.06***	[3.92–43.50]	21.48***	[5.08–90.83]

The adjusted ORs/CIs were adjusted for dummy variables for age categories, Hausa ethnicity, marital status, polygamous status, ever attended school, literacy, birth parity status (parity zero, parity one, and parity two or higher), and assignment to the birth kits arm. The sum of the proportions for the variables facility birth, delivered at home alone, and delivered at home accompanied is less than 100% because 10 respondents are not designated as falling within any of these categories, due to missing data. Asterisks indicate significance at the 5 (*), 1 (**), and .1 (***) percent level.

Table 8 reports parallel specifications analyzing maternal and neonatal morbidity in Panel A and anthropometrics for children under two at endline (i.e., children born during the intervention period) in Panel B. The results indicate that use of a birth kit was associated with increased odds of prolonged labor (aOR 4.75, CI 1.36–16.59, *p* <.05) and postpartum bleeding (aOR 4.10, CI 1.32–12.71, *p* < 0.05). While birth kit use was not otherwise associated with statistically significant differences in morbidity comparing across respondents who do and do not use birth kits, for a number of variables the observed prevalence of complications was higher in the subsample of women who used a birth kit, and thus the odds ratios reported are greater than one. No statistically significant associations were observed between birth kit use and under-two anthropometrics. Given the small sample size, the analysis lacked power to evaluate maternal and neonatal mortality.

**Table 8:** Associations between birth kit use and pregnancy outcomes at the individual level.

	N (%)	N (%)	OR	95% CI	OR	95% CI
	Did not use (n = 68)	Used a kit (n = 72)	Crude	Crude	Adjusted	Adjusted
	**Panel A: Maternal and neonatal morbidity**					
Pregnancy: swelling	25 (36.8)	22 (30.6)	0.78	[0.32–1.90]	0.76	[0.36–1.61]
Pregnancy: fatigue	4 (5.9)	2 (2.8)	5.55	[0.15–205.76]	0.46	[0.05–3.91]
Pregnancy: bleeding	3 (4.4)	2 (2.8)	1.28	[0.19–8.41]	0.62	[0.08–4.96]
Pregnancy: high BP	3 (4.4)	9 (12.5)	13.55	[0.49–377.69]	3.10	[0.78–12.27]
Pregnancy: other	3 (5.3)	5 (7.6)	0.96	[0.28–3.25]	1.48	[0.50–4.32]
Delivery: bleeding	6 (11.3)	5 (7.4)	0.50	[0.15–1.68]	0.62	[0.19–2.02]
Delivery: prolonged labor	4 (7.5)	19 (27.9)	4.87*	[1.19–19.86]	4.75*	[1.36–16.59]
Delivery: headache / blurred vision / high BP	3 (5.7)	4 (5.9)	1.31	[0.23–7.52]	1.04	[0.21–5.23]
Post–partum: bleeding	7 (14.0)	24 (40.0)	4.90*	[1.06–22.67]	4.10*	[1.32–12.71]
Post–partum: swelling	3 (6.1)	4 (6.7)	1.81	[0.10–32.60]	1.10	[0.30–3.95]
Post–partum: fever	6 (12.0)	8 (13.6)	1.90	[0.63–5.78]	1.15	[0.38–3.52]
Post–partum: abdominal pain	3 (6.0)	7 (11.7)	2.60	[0.27–25.04]	2.07	[0.45–9.56]
Neonatal: rash	1 (9.1)	14 (36.8)	27.08	[0.58–1274.75]	5.83	[0.90–37.84]
Neonatal: fever	1 (9.1)	5 (13.2)	1.32	[0.20–8.76]	1.52	[0.12–19.08]
	**Panel B: Under-two anthropometrics**					
Underweight	21 (39.6)	16 (40.0)	1.02	[0.43–2.38]	0.92	[0.36–2.39]
Stunted	21 (39.6)	16 (40.0)	1.02	[0.50–2.05]	0.90	[0.43–1.88]
Low MUAC-for-age	4 (9.5)	8 (21.6)	2.62	[0.72–9.50]	2.39	[0.40–14.3]

The adjusted ORs/CIs were adjusted for dummy variables for age categories, Hausa ethnicity, marital status, polygamous status, ever attended school, literacy, birth parity status (parity zero, parity one, and parity two or higher), and assignment to the birth kits arm, and variables capturing utilization of maternal health care: whether the respondent utilized antenatal care, whether she delivered in a facility, and whether the delivery was attended by a skilled provider. Asterisks indicate significance at the 5 (*), 1 (**), and .1 (***) percent level.

## Discussion

The evidence presented in this analysis suggests that in a northern Nigerian state characterized by minimal utilization of maternal health services, uptake of an intervention designed to distribute safe delivery kits to pregnant women at home was low, and utilization of the kits is not significantly associated with enhanced maternal or newborn health outcomes. In this evaluation, only 9.7% of eligible women received birth kits, a low level of penetration that presumably reflects a number of factors. Operational data from the CoRPs program and qualitative data suggest that performance of the CoRPs was relatively low, reflecting limited training, limited incentives provided to CoRPs, and weak supervision. A high level of insecurity due to ongoing unrest linked to the Boko Haram rebellion also generated challenges for supervision and implementation. A large number of CoRPs were inactive, failing to conduct educational sessions with pregnant women in their homes, and similarly failing to distribute birth kits to eligible pregnant women in their third trimester.

It is challenging to compare the patterns of birth kit receipt and use in this evaluation to previously reported data on birth kit uptake for two reasons. First, previous quantitative studies examining birth kits generally assume that receipt and use of a birth kit are synonymous, and do not report data separately for individuals who received and used kits. Our results suggest that receipt of a kit is not an appropriate proxy for use, as only 50% of women who report receipt of a kit also report use. Second, the existing evidence almost exclusively focuses on interventions in which birth kits are distributed to health facilities, skilled birth attendants, or stores for purchase [12]. There is little existing evidence analyzing how uptake of birth kits may vary with respect to the identity of the recipient (health worker or pregnant woman) [10]. Accordingly, this paper provides a novel contribution to the literature by examining the effects of an intervention that provided kits directly to pregnant women.

In previous literature, reported use of birth kits ranges from 15–100% [10]. Our findings are broadly consistent with a study analyzing data from a cRCT promoting birth kit use in Nepal, India, and Bangladesh: kits were used in 18.4% of home births in India, 18.4% in Bangladesh, and 5.7% in Nepal [17]. However, in that context, birth kits were distributed through the health system, and no data were reported for birth kit receipt vis-a-vis birth kit use. Other evaluations conducted in Tanzania and Egypt have found higher rates of use (60% in Tanzania and 75% in Egypt), but these interventions were more intensive and also relied on distribution via the public health system [14, 15].

Eight qualitative studies have examined women’s experiences with birth kits, the kits’ general acceptability, and recipients’ reasons for use or non-use. This literature has generally reported that delivery and post-natal practices are culturally patterned, and limited knowledge about the kit and perceptions of limited utility are the most important factors constraining use [11]. Other reasons for low use include lack of confidence in using the kit [15, 30], lack of support for its use from family members or traditional birth attendants [15, 30], perceptions that use of the kit is wasteful [31], or simply limited salience of kit receipt [11, 30, 31]. In our setting, data on attitudes towards the kits, perceived utility, or comfort in use were not collected. Our study did, however, assess knowledge and found that conditional on receiving a birth kit, women had relatively high levels of knowledge about the kit’s purpose and contents. This suggests that CoRPs were effectively able to transfer information, and respondents retained this information approximately two years later.

Our findings generally show no statistically significant differences in baseline characteristics or in previous maternal health utilization among women who received a kit versus those who did not receive a kit. This evidence suggests that the CoRPs did not systematically prioritize women with certain characteristics for receipt of a kit, and the pattern is also consistent when the sample is restricted to respondents assigned to the birth kits arm. However, there is evidence that women who used the kits are characterized by better reported health at baseline; they are more likely to use antenatal care, more knowledgeable about general health questions as well as the risks of pregnancy, and more confident that they themselves face minimal risk. Birth kit users are also less likely to report a previous pregnancy complication, and are more likely to report greater involvement by their husbands in a previous pregnancy.

There is some evidence in the literature related to determinants of birth kit use. Evidence from Egypt suggests that women who had higher ANC attendance during the same pregnancy exhibit higher levels of birth kit use [14]. In South Asia, use of a birth kit is negatively associated with low educational levels, positively associated with birth parity, and positively associated with at least one ANC visit [17]. More broadly, a number of characteristics are consistently observed in the literature to be predictive of utilization of antenatal care and maternal services, including maternal education, history of obstetric complications, and household wealth [32]. Maternal education, household wealth and birth parity are not observed to be significantly associated with kit use in this analysis; there is some evidence of a positive association between previous utilization of antenatal care and birth kit use, and a negative association between previous obstetric complications and kit use.

Contrary to other published studies, we find evidence of significant and negative associations between kit use and utilization of formal health care and recommended health practices, as well as maternal health outcomes. Birth kit use was associated with lower odds of receiving four or more ANC visits, but was not significantly associated with utilization of other maternal health services, such as facility based delivery or postnatal care. In addition, there is weak evidence of an association between kit use and increased odds that the husband is present at delivery (though this relationship is statistically significant at the 5 percent level only when adjusted for confounders), and strong evidence of an association between kit use and increased odds of complementary feeding in the first three days. Given that the World Health Organization recommends exclusive breastfeeding in the neonatal period [33], this evidence suggests there may be a correlation between kit use and poor infant feeding practices.

Previous evidence from Balsara et al. suggests that mothers using a safe birth kit were more than four times as likely to seek ANC [14]. However, in that context the kits were provided at ANC visits, and thus kit uptake was tied to ANC attendance; the paper did not report effect on other health services. In a RCT in Zambia, provision of a mama kit containing diapers, a sheet and a blanket to women who deliver at health facilities increased facility-based delivery levels [19]. However, the fact that receipt of the kit was conditional on utilization renders this context very different from the trial analyzed here.

Previous authors have suggested that provision of birth kits could potentially encourage utilization of health services, but given our findings, it appears that this may only be the case when the kit is itself tied to care utilization. Further evidence around the influence of birth kit distribution on health services use is needed to understand differences with respect to the distribution mechanism and the recipient’s characteristics in order to assess and minimize unintended consequences.

Evidence from this study suggesting there is no association between birth kit use and neonatal morbidity and a strong positive correlation between kit use and prolonged labor and postpartum bleeding also differs from previous literature that generally concludes that birth kit use is correlated with reductions in neonatal morbidity and/or associated risk. A number of papers report that birth kit use is associated with a reduction in the risk of cord infection [13, 15, 34] and sepsis [35], as well as enhancement of post-natal infant care practices [14], and reduction in neonatal mortality [17, 36–38]. There is also some evidence that birth kit use is associated with enhanced maternal health outcomes, albeit more limited. A cluster randomized trial in Nepal reported that the distribution of birth kits in conjunction with training of traditional birth attendants led to a reduction in puerperal sepsis and post-partum hemorrhage in the intervention group, as well as increased rates of diagnosis of obstructed delivery and referral to the hospital in case of complications [36]. Several studies reported reduction of puerperal sepsis among women who used a birth kit during delivery [13, 15, 34], but Seward et al. did not find evidence of an association between reported kit use and maternal death [18].

There are several mechanisms that may explain the weak or adverse effects of birth kits observed in this study. First, given the low observed penetration rates of the birth kits intervention, community health workers may have chosen women with certain characteristics to receive birth kits. For example, CoRPs may have targeted the distribution of birth kits to women who are unlikely to use the formal health care system, or women who they perceived to be at higher risk of pregnancy complications. However, our data show almost no evidence of significant correlations between these characteristics as measured at baseline and receipt of a birth kit. Second, women who chose to use the birth kit may have had different characteristics from women who chose not to use the birth kit: for example, kit users may have been higher risk of poor pregnancy outcomes ex ante. However, the observed associations are significant even when adjusted for baseline demographic characteristics. In addition, women who used the birth kits were in general healthier, more informed about pregnancy risk, and perceived themselves to be at lower risk relative to women who did not use the kits. These characteristics are ceteris paribus associated with a higher probability of better health outcomes [39].

A third potential mechanism is that the birth kits may have been viewed as a substitute form of care for women who prefer to avoid utilizing formal care, or who are unable to utilize it. Women who viewed themselves as low risk and women whose spouses were more involved in health care decision-making may have been particularly likely to substitute toward the use of birth kits, assuming that men also have a strong preference to minimize utilization of formal health care. This yields a reduction in the use of formal care, and an increased odds of adverse outcomes. Our data show some evidence that use of birth kits may be correlated with a reduction in use of ANC care, as kit users were less likely to receive ANC via the formal health system or complete four or more ANC visits, but no evidence of substitution away from other types of health services.

A fourth mechanism that would be consistent with the absence of any improvement in neonatal outcomes would be improper use of the birth kit and/or the persistence of other unhygienic or unsafe delivery practices. Previous evaluations that have found positive effects of kit use on health outcomes have also reported increased hand washing and other clean delivery practices [14, 18]. Unfortunately, data on other unhygienic practices during the delivery or in the postpartum period were not available.

This study has several strengths. First, it utilizes a unique data set collected at multiple points in time from a population characterized by extremely low levels of human capital and poor health outcomes. Second, participants were randomly selected within communities, ensuring a representative sample. Third, separate data was collected about exposure to the intervention (birth kit receipt) and use of the birth kit, as well as intermediate outcomes such as knowledge, allowing us to identify gaps between receipt of the birth kit and use.

However, this study also has several limitations. The analysis utilizes a relatively small sample of birth kit recipients, limiting statistical power for the analysis of the effects of use on health outcomes. Data about care utilization during pregnancy and pregnancy outcomes were collected from respondents at different points in time, and recall bias may be a challenge for respondents who were surveyed only at endline.

In addition, given that this is a secondary analysis of cRCT data rather than an ITT analysis, the paper presents only correlational evidence related to birth kit use [40, 41]. While this analysis cannot generate causal conclusions, the descriptive statistics suggest that in this setting, there was a substantial gap between the goal of the intervention (all pregnant women in treatment communities were eligible to receive birth kits), programmatic execution, and beneficiary utilization. Given that these gaps are not uncommon in developing countries, analyzing correlational evidence about the effects of this variation in execution and utilization is nonetheless informative.

Given that the sample was drawn from one state in northern Nigeria, the evidence presented here may have limited external validity for a broader population. However, this evidence may be relevant for other similar populations (particularly rural areas characterized by extremely low baseline utilization rates of health services and poor health outcomes for women and children), as well as for other interventions in which community health workers characterized by low levels of training and/or supervision promote new health technologies or health inputs.

Our findings have implications for health programming, policy and research. While further research is needed to understand how the effectiveness of birth kits is shaped by the distribution mechanism and the characteristics of recipients, this evidence suggests that the provision of kits to pregnant women outside of the formal health system may be undesirable. Use of birth kits may encourage substitution away from the formal health system (at least for ANC), and appears to be associated with negative health outcomes. Distribution via skilled health workers or linking distribution to health service utilization may have more positive effects on beneficiaries; however, this choice also has implications for cost-effectiveness.

In light of the growing literature suggesting that safe birth kits are effective in enhancing clean delivery practices and reducing maternal and neonatal health risks, it is important to highlight that the benefits of birth kits may not be universal. Future interventions developed for settings where utilization of formal health care is particularly low should take into account the potential risk that birth kits distribution may in fact reinforce a preexisting preference to avoid utilizing care and contribute to poor health outcomes. Further evidence is needed to understand whether birth kit distribution dis-incentivizes facility delivery in different contexts.

## Conclusion

Our findings suggest that an intervention in rural northern Nigeria designed to distribute safe birth kits widely to all pregnant women in targeted communities resulted in relatively low rates of penetration, and even lower rates of birth kit use. In this setting, the use of birth kits was not associated with reductions in maternal or neonatal morbidity, but rather seems to be associated with an increase in adverse health outcomes. Further research should explore the potential risks of birth kit distribution in reinforcing low utilization rates of formal health care, particularly in contexts where resistance to formal health services is relatively high.
